# Clopidogrel with indobufen or aspirin in minor ischemic stroke or high-risk transient ischemic attack: a randomized controlled clinical study

**DOI:** 10.1186/s12883-024-03585-4

**Published:** 2024-03-01

**Authors:** Xudong Liu, Xuxian Lv, Yanfang Peng, Jianing Wang, Junjie Lei, Chaogang Tang, Shijian Luo, Weihua Mai, Yiming Cai, Qian Fan, Chenhao Liu, Lei Zhang

**Affiliations:** The Fifth Affiliated Hospital of Sun Yat-sen University, No. 52 Meihua East Road, Zhuhai City, Guangdong Province China

**Keywords:** Acute ischemic stroke, High-risk TIA, Dual antiplatelet, Recurrence rate, Bleeding events

## Abstract

**Background:**

Ischemic stroke and transient ischemic attack (TIA) are the most prevalent cerebrovascular diseases. The conventional antiplatelet drugs are associated with an inherent bleeding risk, while indobufen is a new antiplatelet drug and has the similar mechanism of antiplatelet aggregation as aspirin with more safety profile. However, there have been no studies evaluating the combination therapy of indobufen and clopidogrel for antiplatelet therapy in cerebrovascular diseases.

**Objective:**

The CARMIA study aims to investigate the effectiveness and safety of a new dual antiplatelet therapy consisting of indobufen and clopidogrel comparing with the conventional dual antiplatelet therapy consisting of aspirin and clopidogrel in patients with minor ischemic stroke or high-risk TIA.

**Methods:**

An open-label randomized controlled clinical trial was conducted at a clinical center. We randomly assigned patients who had experienced a minor stroke or transient ischemic attack (TIA) within 72 h of onset, or within 1 month if they had intracranial stenosis (IS), to receive either indobufen 100 mg twice daily or aspirin 100 mg once daily for 21 days. For patients with IS, the treatment duration was extended to 3 months. All patients received a loading dose of 300 mg clopidogrel orally on the first day, followed by 75 mg once daily from the second day to 1 year. We collected prospective data using paper-based case report forms, and followed up on enrolled patients was conducted to assess the incidence of recurrent ischemic stroke or TIA, mRS score, NIHSS (National Institutes of Health Stroke Scale) score, and any bleeding events occurring within 3 month after onset.

**Results:**

We enrolled 202 patients diagnosed with ischemic stroke or transient ischemic attack. After applying the criteria, 182 patients were eligible for data analysis. Endpoint events (recurrence of ischemic stroke/TIA, myocardial infarction, or death) were observed in 6 patients (6.5%) receiving aspirin and clopidogrel, including 4 (4.3%) with stroke recurrence, 1 (1.1%) with TIA recurrence, and 1 (1%) with death. In contrast, no endpoint events were reported in the indobufen and clopidogrel group (*P* = 0.029). The group of patients receiving indobufen and clopidogrel exhibited significantly lower modified Rankin Scale (mRS) score. (scores range from 0 to 6, with higher scores indicating more severe disability) compared to the aspirin and clopidogrel group (common odds ratio 3.629, 95% CI 1.874–7.036, *P* < 0.0001). Although the improvement rate of NIHSS score in the indobufen and clopidogrel group was higher than that in the aspirin and clopidogrel group, the difference was not statistically significant (*P* > 0.05). Bleeding events were observed in 8 patients (8.6%) receiving aspirin and clopidogrel, including 4 (4.3%) with skin bleeding, 2 (2.2%) with gingival bleeding, 1 (1.1%) with gastrointestinal bleeding, and 1 (1.1%) with urinary system bleeding. On the other hand, only 1 patient (1.1%) in the indobufen and clopidogrel group experienced skin bleeding (*P* = 0.035).

**Conclusion:**

The combination of indobufen and clopidogrel has shown non-inferior and potentially superior effectiveness and safety compared to aspirin combined with clopidogrel in patients with minor ischemic stroke and high-risk TIA in the CARMIA study (registered under chictr.org.cn with registration number ChiCTR2100043087 in 01/02/2021).

## Key messages

**What is already known on this topic:**

Indobufen is comparable to aspirin in the treatment of atherosclerotic ischemic heart and peripheral vascular diseases, but the effect on minor ischemic stroke or high-risk TIA secondary prevention is unclear.

**What this study adds:**

The Randomized Controlled Clinical Study of Clopidogrel with Indobufen or Aspirin in Minor Ischemic Stroke or High-risk Transient Ischemic Attack suggests that combination of indobufen and clopidogrel has demonstrated superior effectiveness and safety compared to aspirin combined with clopidogrel in patients with minor ischemic stroke and high-risk TIA. Our trial results suggest that initiating dual antiplatelet therapy within 72 h is still beneficial for the patients with minor stroke and high-risk TIA, suggesting the possibility of expanding the time window of dual antiplatelet therapy.

**How this study might affect research, practice or policy:**

The result of the trial may provide a better dual antiplatelet therapeutic regimen for minor ischemic stroke or high-risk TIA secondary prevention.

## Introduction

Ischemic stroke and transient ischemic attack (TIA) are the prevailing types of cerebrovascular disease. In China, ischemic stroke is the leading cause of disability and death, with approximately 30% of cases classified as minor strokes [1, 2]. TIA is an independent risk factor for ischemic stroke, especially the high-risk TIA.

Aspirin in combination with clopidogrel is the standard antiplatelet therapy for minor stroke and high-risk TIA. Nonetheless, it increases the risk of bleeding, mostly in the gastrointestinal tract. Besides, aspirin resistance, a phenomenon in which the low physiological reactivity of aspirin reduces its antiplatelet effect, has been observed in 5–45% of the population [3]. Indobufen is a new antiplatelet drug and has a mechanism of antiplatelet aggregation similar to that of aspirin. Some studies have demonstrated that the effectiveness of indobufen is equivalent to aspirin, and with a superior safety profile [4]. Meanwhile, there is no evidence of indobufen resistance. So indobufen might be a promising candidate for combination therapy with clopidogrel. However, no study has yet evaluated the combination therapy of indobufen and clopidogrel for antiplatelet therapy in cerebrovascular diseases.

The aim of this study is to investigate whether the combination of indobufen with clopidogrel is more effective and safer compared to the conventional aspirin combined with clopidogrel antiplatelet therapy in minor stroke and high-risk TIA.

## Methods

### Study oversihght

This study was conducted in adherence to a pre-defined protocol and statistical analysis plan, developed by the researchers and overseen by GCP with unrestricted access to the data. The authors assume full responsibility for the data’s accuracy, completeness, and the report’s adherence to the study protocol. No commercial support was received for this study. Written informed consent was obtained from all participants or their legal representatives. The CARMIA protocol was approved by the Ethics Committee of the Fifth Affiliated Hospital of Sun Yat-sen University.

### Study population

Patients aged 18 years or older were recruited, Inclusion criteria included: First onset; Acute non-cardioembolic mild ischemic stroke patients (NIHSS score ≤ 3) or TIA with high risk of stroke recurrence (ABCD2 score ≥ 4) within 72 h of onset, or ischemic stroke or TIA patients with symptomatic severe stenosis (stenosis rate of 70%∼99%) in intracranial arteries within 1 month of onset; Patients or their family members signed an informed consent form.

Exclusion criteria included: Ischemic cerebrovascular disease caused by non-atherosclerotic conditions such as vascular malformations, tumors, abscesses, or other non-atherosclerotic conditions; Isolated sensory symptoms (such as numbness), isolated visual changes, or isolated dizziness or vertigo without evidence of acute infarction on baseline CT or MRI of the head; An initial modified Rankin Scale (mRS) score exceeding 2 points before the occurrence of acute ischemic stroke or transient ischemic attack; Clear indication for anticoagulation therapy (assuming cardiac source of embolus, such as atrial fibrillation or prosthetic heart valves); History of intracranial hemorrhage; anticipated need for long-term non-study antiplatelet drugs or non-steroidal anti-inflammatory drugs that affect platelet function; Heparin therapy or oral anticoagulation therapy within 10 days before randomization; Gastrointestinal bleeding or major surgery within the previous 3 months; Planned or potential vascular reconstruction (any angioplasty or vascular surgery) within 3 months after screening; Plan for surgery or interventional treatment that requires discontinuation of the study drug; TIA or minor stroke caused by angiography or surgery; Severe non-cardiovascular coexisting diseases with a life expectancy of less than 3 months; Women of childbearing age who have not taken reliable contraceptive measures, have not had a negative pregnancy test on record, and patients receiving other study drugs or devices; Contraindications to indobufen, aspirin, or clopidogrel.

Termination criteria included: The patient refuses to continue the study; If serious complications and toxic side effects occur, the investigator considers that it is medically necessary to terminate the treatment for the subject.

### Study design

CARMIA is a prospective, open-label, randomized, controlled clinical trial conducted at a single center. The study included a total of 202 patients who met the inclusion criteria and were randomly assigned to the experimental group, which received indobufen and clopidogrel, or the control group, which received aspirin and clopidogrel, using a random control table method. The trial consisted of two stages, with the first stage involving 30 patients to confirm safety, while the remaining 172 patients were observed in the second stage. In the experimental group, patients with acute non-cardiogenic TIA with a high risk of stroke recurrence within 72 h (ABCD2 score ≥ 4) or minor ischemic stroke (NIHSS score ≤ 3) were administered indobufen 100 mg orally twice a day and a loading dose of clopidogrel 300 mg orally once daily on the first day, followed by indobufen 100 mg orally twice a day and clopidogrel 75 mg orally once daily from the second day to the 21st day, and clopidogrel 75 mg orally once daily from the 22nd day to 1 year. Patients with symptomatic severe intracranial arterial stenosis (stenosis rate 70%∼99%) within one month of onset were given indobufen 100 mg orally twice a day and a loading dose of clopidogrel 300 mg orally once daily on the first day, followed by indobufen 100 mg orally twice a day and clopidogrel 75 mg orally once daily from the second day to the 3rd month, and clopidogrel 75 mg orally once daily from the 4th month to 1 year. In the control group, patients with acute non-cardiogenic TIA or minor ischemic stroke (NIHSS score ≤ 3) and a high risk of stroke recurrence within 72 h (ABCD2 score ≥ 4) were administered aspirin 100 mg orally once daily and a loading dose of clopidogrel 300 mg orally once daily on the first day, followed by aspirin 100 mg orally once a day and clopidogrel 75 mg orally once daily from the second day to the 21st day, and clopidogrel 75 mg orally once daily from the 22nd day to 1 year. Patients with symptomatic severe intracranial arterial stenosis (stenosis rate 70%∼99%) within one month of onset were given aspirin 100 mg orally once a day and a loading dose of clopidogrel 300 mg orally once daily on the first day, followed by aspirin 100 mg orally once a day and clopidogrel 75 mg orally once daily from the second day to the 3rd month, and clopidogrel 75 mg orally once daily from the 4th month to 1 year. The management of bleeding in patients receiving antiplatelet treatment was dependent on the severity of bleeding. Additional interventions were based on the “Chinese Guidelines for the Diagnosis and Treatment of Acute Ischemic Stroke” and included routine measures such as intravenous thrombolysis, maintenance of electrolyte balance, oxygen therapy, lipid regulation, blood sugar control, blood pressure control, brain cell protection, and prevention of infection.

### Study drugs

Aspirin (Bayer AG, National Drug Approval Number J20171021) 100 mg; Clopidogrel (Sanofi-Aventis Hangzhou Pharmaceutical Co., Ltd., National Drug Approval Number J20080090) 75 mg; Indobufen (Hangzhou Zhongmei Huadong Pharmaceutical Co., Ltd., National Drug Approval Number H20163311) 100 mg. The aforementioned companies had no other role in the study.

### Study outcomes

Primary effectiveness outcomes: composite events (recurrence rate of ischemic stroke or TIA, myocardial infarction, and death) and mRS at 3 months. (The mRS scores range from 0 to 6, with higher scores indicating more severe disability.) Primary safety outcomes: Incidence of bleeding events within 3 months, including gastrointestinal bleeding, intracranial hemorrhage, gingival bleeding, skin bleeding, and urogenital bleeding. Secondary effectiveness outcomes: Improvement rate in NIHSS scores at 3 months compared to baseline.

### Statistic

Sample Size Calculation: This study is designed as a randomized controlled non-inferiority clinical trial, with the aim of assessing the effectiveness and safety of a novel dual antibody combination in preventing ischemic stroke and transient ischemic attack (TIA) recurrence. Based on previous literature reporting a stroke recurrence rate of 8.2% [5] in patients treated with aspirin and clopidogrel, we anticipated that the new combination therapy will yield a recurrence rate of ≤ 8.2%. A two-sided test with a significance level of α = 0.05 and a power of 1-β = 0.9 will be employed, and the statistical analysis will be performed using the χ^2^ test. By utilizing G*power (3.1.92) software, we have calculated that a total of 183 patients will be required to detect significant differences in treatment effects with a 90% probability. To account for a potential dropout rate of 10%, a total of 202 patients will be enrolled in the study.

Statistical Analysis: The data collected from this study will be analyzed using SPSS25.0 statistical software. The mean ± standard deviation (x ± s) will be used to express metric data, and t-tests will be utilized for data analysis. Count data will be analyzed using the χ^2^ test. A *P*-value of less than 0.05 will be considered statistically significant.

## Results

### Study patients and follow-up

**Fig. 1:**
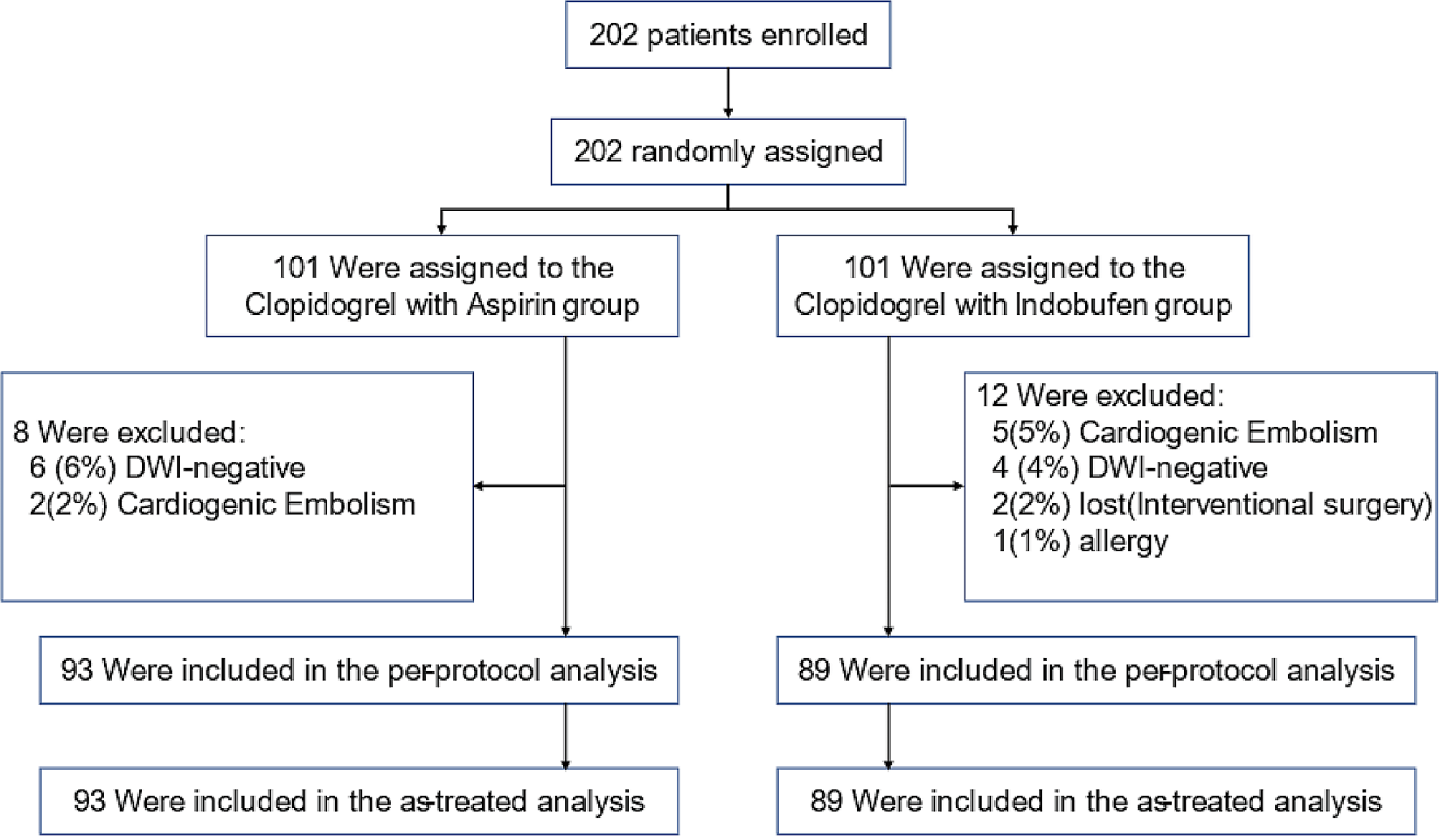
Screening, Randomization, and Follow-up of the Patients

Between February 2021 and February 2023, the Department of Cerebrovascular Disease at the Fifth Affiliated Hospital of Sun Yat-sen University enrolled 202 patients diagnosed with ischemic stroke or transient ischemic attack. In a safety analysis encompassing 30 patients, no endpoint events were detected, thus affirming the study’s safety profile. Following this assessment, the investigation proceeded with an additional cohort of 172 patients. 182 patients met the criteria, and we conducted data analysis on them. Of these patients, 101 were randomly allocated to receive clopidogrel plus aspirin, but 8 were subsequently excluded from the group due to DWI negativity (6%) or cardioembolic stroke (2%). Similarly, 101 patients were assigned to the indobufen plus clopidogrel group, but 12 were excluded from the group due to cardioembolic stroke (5%), DWI negativity (4%), lost (Interventional surgery) (2%), or allergic drug withdrawal (1%). (Fig. 1)

The results of baseline characteristics of the patients demonstrated no significant intergroup differences in gender, age, BMI, systolic and diastolic blood pressure, hypertension, coronary heart disease, hyperlipidemia, diabetes, arrhythmia, smoking and drinking history, TOAST classification (Trial of Org 10,172 in Acute Stroke Treatment), WBC (White Blood Cell Count), ESR (Erythrocyte Sedimentation Rate), CRP (C-reactive Protein), TG (Triglyceride), HDL-C (High Density Lipoprotein Cholesterol), LDL-C (Low Density Lipoprotein Cholesterol), FIB (Fibrinogen), GLU (Glucose), HbA1c (Glycosylated Hemoglobin, Type A1C), or tHcy (Total Homocysteine) (*P* > 0.05). These findings suggest that the baseline characteristics of the two groups were well-balanced. (Table 1)

**Table 1:** Baseline characteristics of the patients

Characteristic	Clopidogrel with Aspirin(*n* = 93)	Clopidogrel with Indobufen(*n* = 89)	t or χ^2^	*P*
Female sex -no. (%)	73 (78.5)	61 (67.8)	χ^2^ = 2.321	0.128
Age (yr)	60.8 ± 12.3	60.8 ± 11.5	t=-0.006	0.995
Interquartile range	53–71	52–68		
BMI (kg/m^2^)	24.7 ± 3.15	25.6 ± 2.64	t = 1.915	0.060
Systolic pressure (mmHg)	146 ± 21	144 ± 18	t = 0.833	0.407
Interquartile range	132–160	132–154		
Diastolic pressure (mmHg)	88 ± 14	91 ± 13	t=-1.370	0.174
Interquartile range	80–98	80–101		
Medical history -no. (%)				
Hypertension	60 (64.5)	62 (69.7)	χ^2^ = 0.545	0.460
Coronary heart disease	5 (5.4)	4 (4.5)	-	1.000
Hypercholesterolemia	3 (3.2)	4 (4.5)	-	0.716
Diabetes mellitus	16 (17.2)	12 (13.5)	χ^2^ = 0.484	0.487
Arrhythmia	0 (0)	0 (0)	-	-
Current or previous smoking - no. (%)	51 (54.8)	43 (48.3)	χ^2^ = 0.775	0.379
Current or previous alcohol - no. (%)	32 (34.4)	31 (34.8)	χ^2^ = 0.004	0.952
TIA	4 (4.3)	4 (4.4)	χ^2^ = 0.002	0.962
ABCD2 Score	4	4.25		
Minor stroke	89	85	-	1.000
TOAST -no. (%)				
Large-artery Atherosclerosis (LAA)	38 (42.7)	26 (30.6)	χ^2^ = 2.741	0.098
Small-Artery Occlusion (SAO)	46 (51.2)	53 (62.3)	χ^2^ = 2.017	0.156
Stroke of Other Determined Causes (SOE)	1 (1.1)	0 (0)	-	1.000
Stroke of Undetermined Etiology (SUE)	4 (4.5)	6 (7.0)	χ^2^ = 0.161	0.689
WBC (×10^9^/L)	7.84 ± 2.43	7.5 ± 2.38	t = 0.902	0.370
ESR (mm/H)	15.8 ± 13.88	13.1 ± 14.83	t = 0.752	0.458
CRP (mg/L)	4.28 ± 5.80	3.84 ± 7.25	t = 0.374	0.710
TG (mmol/l)	1.96 ± 1.56	1.86 ± 1.10	t = 0.468	0.641
TC (mmol/l)	5.11 ± 1.44	4.95 ± 1.01	t = 0.926	0.357
HDL-C (mmol/l)	1.09 ± 0.32	1.18 ± 0.47	t=-1.415	0.161
LDL-C (mmol/l)	3.13 ± 1.01	3.03 ± 0.96	t = 0.855	0.395
FIB	3.13 ± 0.85	3.05 ± 0.81	t = 0.697	0.488
GLU (mmol/l)	6.42 ± 2.87	6.21 ± 2.60	t = 0.453	0.652
HbA1c (%)	6.51 ± 1.57	6.55 ± 1.54	t=-0.211	0.833
tHcy (mmol/l)	15.89 ± 12.34	13.60 ± 4.05	t = 1.630	0.107

Abbreviation: TOAST classification, Trial of Org 10,172 in Acute Stroke Treatment; WBC, White Blood Cell Count; ESR, Erythrocyte Sedimentation Rate; CRP, C-reactive Protein; TG, Triglyceride; HDL-C, High Density Lipoprotein Cholesterol; LDL-C, Low Density Lipoprotein Cholesterol, FIB, Fibrinogen; GLU, Glucose; HbA1c, Glycosylated Hemoglobin, Type A1C; tHcy, Total Homocysteine

### Primary outcomes

**Fig. 2:**
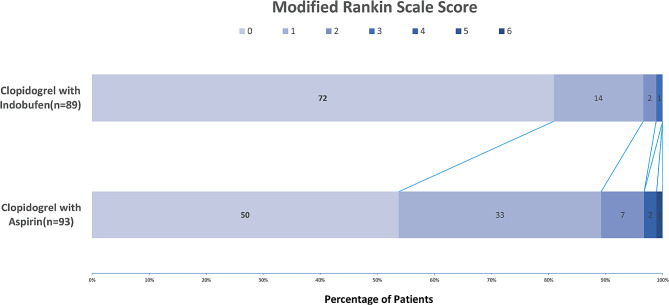
Distribution of modified Rankin Scale(mRS) score at 90 days in CARMIA trial. The mRS category 3,5 for the CARMIA Clopidogrel with Aspirin and 4,5,6 for the Clopidogrel with Indobufen is none and therefore not visible in the figure

The present study aimed to evaluate the effectiveness of a new combination of dual antibodies in preventing stroke recurrence and improving functional outcomes. The primary effectiveness outcomes were assessed using the composite ending events (included stroke or TIA recurrence, myocardial infarction, and death) and mRS score at 3 months. there were no composite endpoint events in the indobufen plus clopidogrel group, while there were the aspirin plus clopidogrel group had 6 patients (6.5%) with composite endpoint events in the aspirin plus clopidogrel group, including 4 (4.3%) with stroke recurrence, 1 (1.1%) with TIA recurrence, and 1 (1%) death. A multivariate ordinal logistic regression analysis (shift analysis) was employed to express the impact on the mRS score as an adjusted common odds ratio. Furthermore, The mRS score of the indobufen plus clopidogrel group was found to be significantly better than that of the aspirin plus clopidogrel group, with a statistically significant difference (common odds ratio 3.629, 95% CI 1.874–7.036, *P* < 0.0001). These findings suggest that the new dual antibody combination is a more effective treatment for preventing stroke recurrence and improving functional outcomes than the traditional combination of aspirin and clopidogrel. (Table 2; Fig. 2)

**Table 2:** Trial primary effectiveness outcomes

Primary effectiveness outcomes	Clopidogrel with Aspirin (*n* = 93)	Clopidogrel with Indobufen (*n* = 89)	Effect Variable	Value (95% CI)	*p*
Ending event(%)	6(6.5)	0(0)	Hazard ratio	0.94(0.89–0.99)	0.029
Modified Rankin scale score at 90 days - IQR	1(1–2)	0(0–0)	Common odds ratio	3.629(1.874–7.036)	< 0.0001

**Fig. 3:**
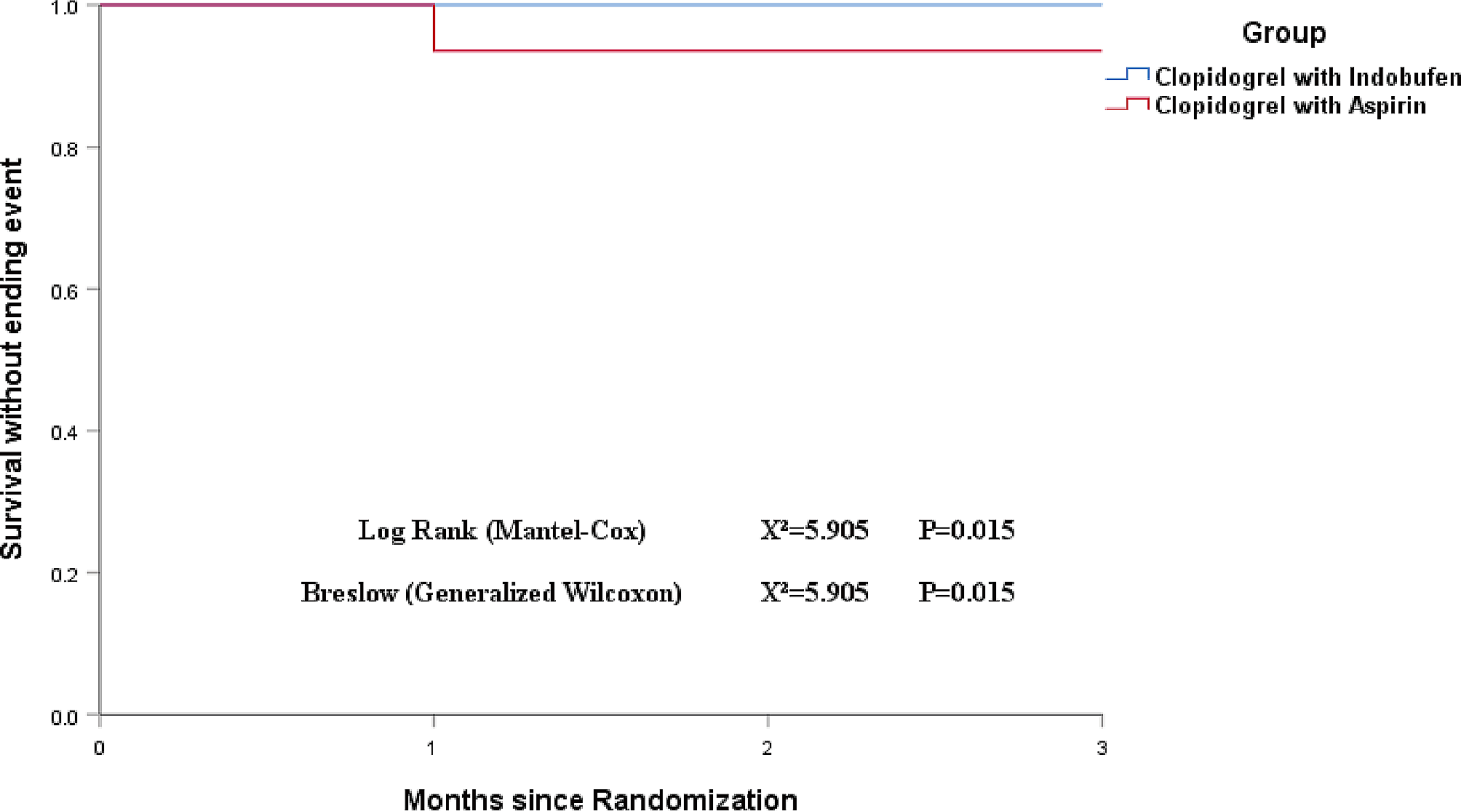
Probability of survival without ending event

The Kaplan-Meier curve analysis demonstrated a significant difference (*P* = 0.015) in the occurrence of endpoint events between the group treated with indobufen plus clopidogrel and the group treated with aspirin plus clopidogrel. The results indicate that the indobufen plus clopidogrel treatment was associated with better outcomes than the aspirin plus clopidogrel treatment. (Fig. 3)

The primary safety outcome in this study was the occurrence of major bleeding events, encompassing various types of bleeding such as gastrointestinal bleeding, intracranial bleeding, gingival bleeding, skin bleeding, and genitourinary bleeding. Out of the 101 patients in the aspirin plus clopidogrel group, 8 patients (8.6%) experienced bleeding events, with 4 cases (4.3%) of skin bleeding, 2 cases (2.2%) of gingival bleeding, 1 case (1.1%) of gastrointestinal bleeding and 1 case (1.1%) of genitourinary bleeding. In contrast, only 1 patient (1.1%) in the indobufen plus clopidogrel group experienced a bleeding event, which was skin bleeding. The difference of the incidence of bleeding events between the two groups was statistically significant (*P* = 0.035). (Table 3)

**Table 3:** Trial primary safety outcome

Primary safety outcome	Clopidogrel with Aspirin (*n* = 93)	Clopidogrel with Indobufen (*n* = 89)	χ2	Hazard Ratio (95% CI)	*P*
Bleeding(%)	8(8.6)	1(1.1)	-	0.92 (0.87–0.99)	0.035

**Fig. 4:**
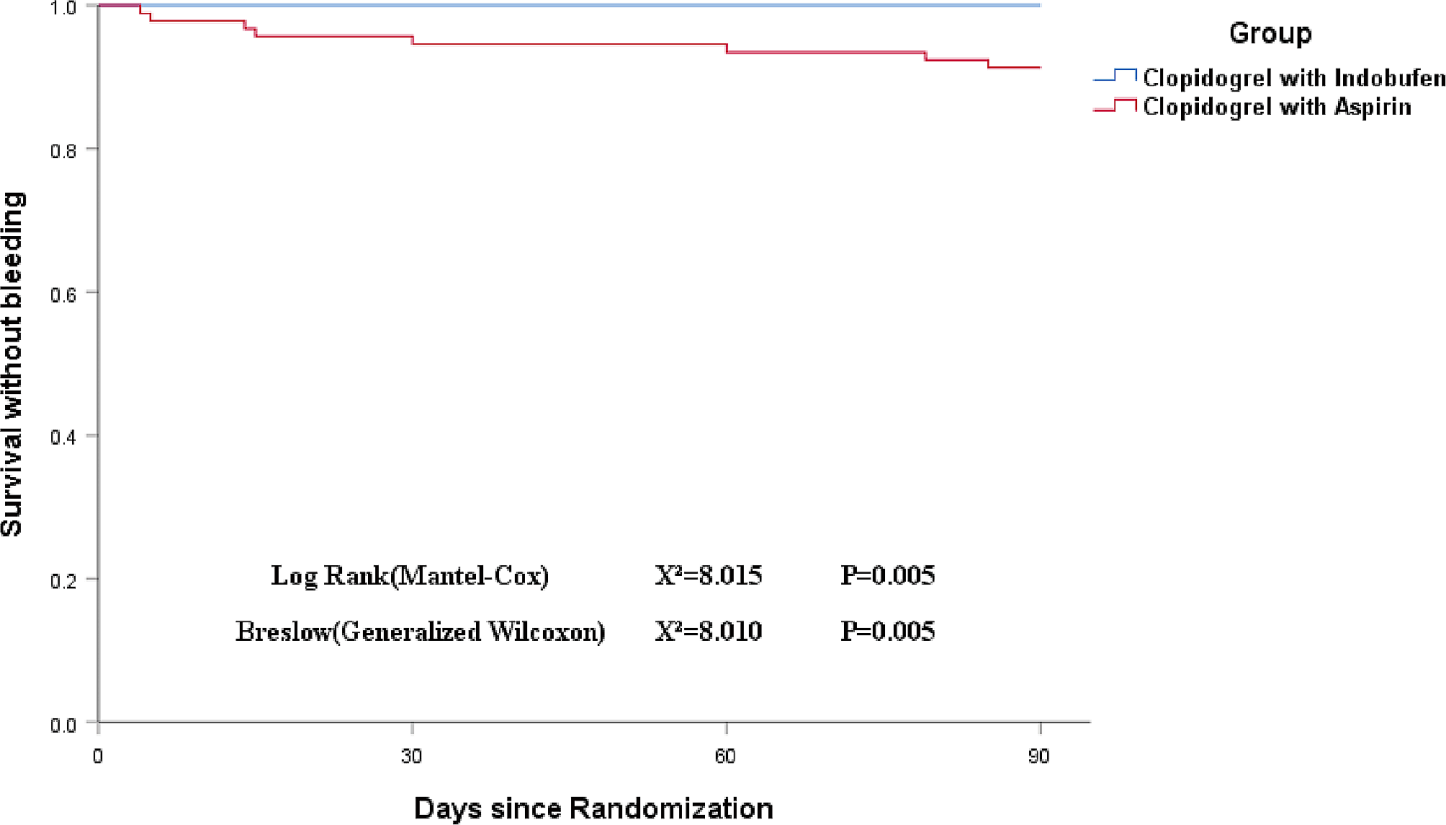
Probability of survival without bleeding

The Kaplan-Meier curves for bleeding events demonstrated a significant difference between the indobufen plus clopidogrel group and the aspirin plus clopidogrel group, with the former exhibiting a clear advantage (*P* = 0.005). (Fig. 4).

### Secondary outcome

The study’s secondary outcome was to assess the difference in NIHSS scores between baseline and three months post-treatment. The ∆NIHSS score of the indobufen plus clopidogrel group was compared to that of the aspirin plus clopidogrel group. While the results showed that the indobufen plus clopidogrel group had a marginally higher ∆NIHSS score compared to the aspirin plus clopidogrel group (*P* = 0.123). (Table 4)

**Table 4:** Trial secondary outcome

Secondary effectiveness outcome	Clopidogrel with Aspirin (*n* = 93)	Clopidogrel with Indobufen (*n* = 89)	t	*P*
∆NHISS	1.34 ± 1.74	1.71 ± 1.40	t =-1.557	0.123

**Fig. 5:**
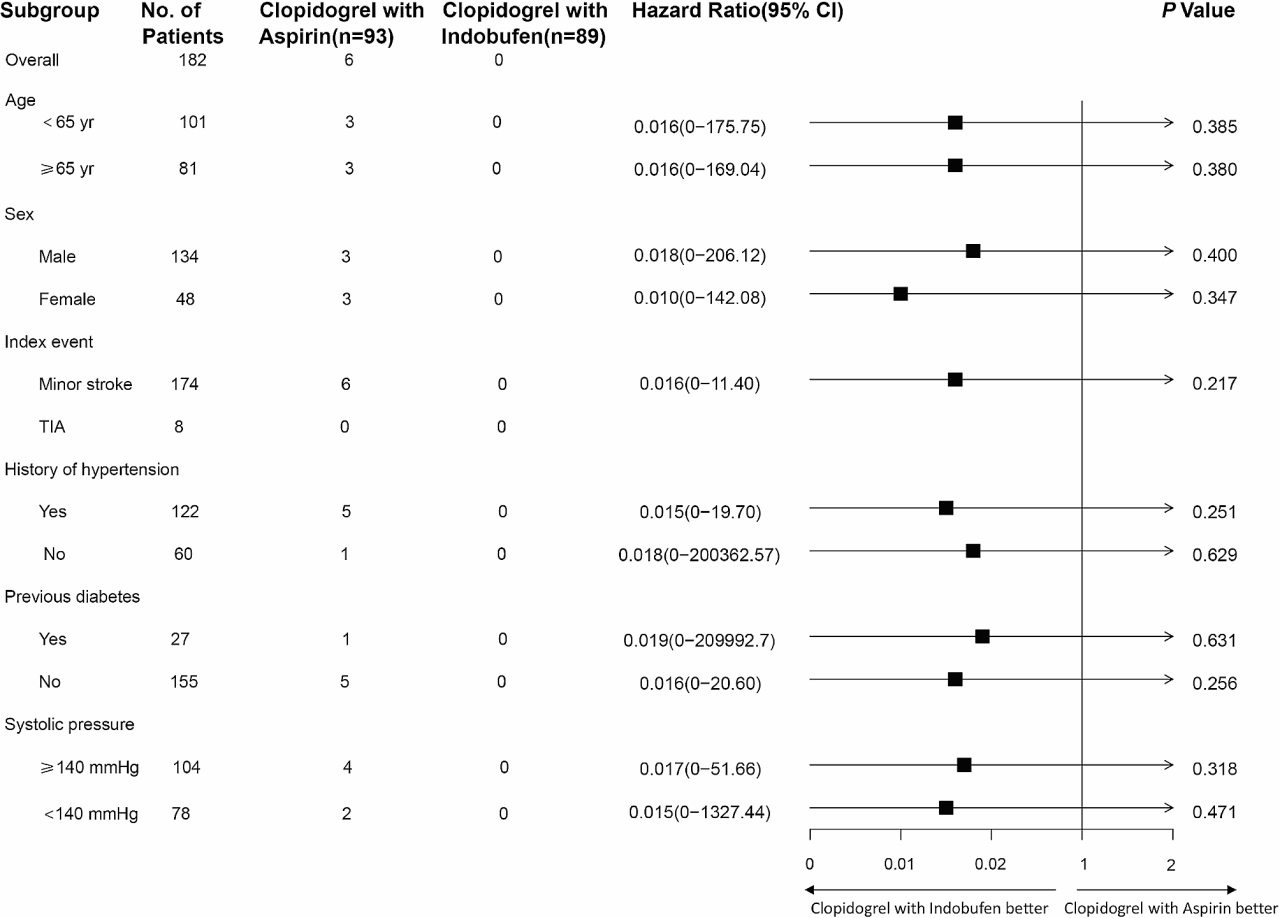
Hazard ratio for the primary outcome in prespecified subgroups. In the pre-specified subgroups. compared with aspirin combined with clopidogrel the reduction in stroke risk with indobufen combined with clopidogrel was consistent across all major subgroups. In six pre-defined subgroups, there was no significant interaction in any of the subgroups (all comparisons with *P* > 0.10)

The findings suggest that the decreased stroke risk associated with indobufen plus clopidogrel compared with aspirin plus clopidogrel is consistent among all major subgroups identified prior to the study. Analysis of the 6 pre-specified subgroups revealed no significant interaction in any subgroup, with all *P*-values exceeding 0.10. These results suggest that the treatment effect observed in the overall population is not significantly affected by any of the pre-defined subgroups. (Fig. 5)

## Discussion

In this clinical trial involving patients with minor stroke and high-risk TIA, we found that the combination of indobufen and clopidogrel reduced the risk of subsequent stroke by 6.0%compared to traditional aspirin combined with clopidogrel. Previous studies such as CHANCE, POINT, and CLAIR [5–7] have demonstrated the effectiveness of aspirin and clopidogrel dual antiplatelet therapy in reducing the risk of stroke recurrence compared to aspirin alone, but with an increased risk of bleeding. We newly proposed that dual antiplatelet therapy with indobufen and clopidogrel reduces the incidence of bleeding events by 8% compared to traditional aspirin and clopidogrel therapy.

The findings of our clinical trial suggest that the dual antiplatelet therapy of aspirin and clopidogrel is less effective than the combination of indobufen and clopidogrel for patients with minor stroke and high-risk TIA. This could be attributed to the phenomenon of aspirin resistance, which is prevalent among Asian populations and results in reduced effectiveness of aspirin in preventing atherosclerotic events [3, 8, 9]. Besides, indobufen has been shown to have a superior antiplatelet effect than aspirin in many studies and no resistance has been reported [4, 10]. Furthermore, indobufen possesses anticoagulant properties and provides a more comprehensive approach to treating stroke caused by multiple etiologies, such as perforating artery disease. Compared to unfractionated heparin and low molecular weight heparin, indobufen is more effective [11, 12], so it might reduce deep vein thrombosis which is a common complication in stroke.

Regarding safety, our findings suggested that the combination of indobufen and clopidogrel is superior to the combination of clopidogrel and aspirin. One possible explanation is that the antiplatelet effect of aspirin is irreversible and lasts for approximately 7 days, whereas the effect of indobufen is short-lived, and normal platelet function is restored within 24 h after discontinuation [13]. Existing literature indicated that indobufen has a lower bleeding risk and is more controllable, compared to aspirin [13]. Furthermore, indobufen has been shown to have a lower incidence of gastrointestinal adverse reactions and better tolerance than aspirin, leading to better adherence [14, 15]. As evidenced by previous literature, aspirin-associated bleeding events are commonly seen in the gastrointestinal tract with an incidence rate of 1.5% per annum [16, 17]. However, contrary to these findings, our clinical investigation revealed that skin bleeding was the most prevalent form of hemorrhage. Patients may be more sensitive to detecting cutaneous bleeding events compared to mild gastrointestinal bleeding, which may go unnoticed unless it progresses to a severe stage. Furthermore, the majority of gastrointestinal bleeding cases induced by aspirin are of mild severity. Another factor contributing to the decreased occurrence of gastrointestinal bleeding was the utilization of rabeprazole in tandem with dual antiplatelet therapy, as a prophylactic measure to safeguard the gastrointestinal tract. Additionally, the small sample size is also one of the reasons for the low incidence of gastrointestinal bleeding.

In the most recent OPTION (Indobufen or Aspirin on Top of Clopidogrel After Coronary Drug-Eluting Stent Implantation) study, it was found that the effectiveness and safety composite endpoint at 1 year with indobufen plus clopidogrel dual antiplatelet therapy (DAPT) for 12 months was non-inferior to aspirin plus clopidogrel DAPT [18]. The indobufen plus clopidogrel DAPT showed no significant difference from conventional DAPT in the 1-year effectiveness composite endpoint, including cardiovascular death, nonfatal myocardial infarction, ischemic stroke, and definite or probable stent thrombosis, while it reduced the incidence of bleeding events, most of which were mild. This finding is in line with the results of our trial and further supports the feasibility of using indobufen with clopidogrel DAPT.

Genetic testing to guide precise secondary prevention antiplatelet therapy could address the issue of aspirin resistance, but its high cost and low speed of producing results renders it impractical as a routine diagnostic and treatment approach. In light of the aforementioned limitations, we were unable to perform aspirin resistance testing on the control group patients enrolled in our study to substantiate our findings. According to the previous researches of aspirin resistance, we hypothesize that reduced effectiveness of aspirin treatment could potentially, at least partially be attributed to aspirin resistance.

According to guidelines, dual antiplatelet therapy is recommended for minor stroke and high-risk TIA patients within 24 h of onset. This study extended the treatment window to 72 hours [19]. The acute phase of stroke is known to last for the first week after onset, during which the risk of recurrence of the stroke is high [20]. Therefore, we tried to explored the initiation time of dual antiplatelet therapy in this study. Our trial results suggest that initiating dual antiplatelet therapy within 72 h is still beneficial for the patients with minor stroke and high-risk TIA, suggesting the possibility of expanding the time window of DAPT.

This study presents a randomized controlled clinical trial conducted at a single center, which enrolled 202 patients with stroke or high-risk TIA. The trial’s design was limited by factors such as a short recruitment period, inadequate personnel, and a small sample size. Blinding was not feasible during patient enrollment and drug administration; however, the evaluation of outcomes was conducted by evaluators who were blinded to the treatment conditions. Given the restricted sample size in our investigation, the inadequacy of samples within subgroups poses a challenge to the credibility of our results. Accordingly, we envisage undertaking subgroup analyses within a broader, multicenter study to ensure more robust and reliable findings. It is just preliminary exploratory research.

Following the trial, an extended follow-up will be undertaken to acquire more extensive clinical data, enabling a thorough evaluation of long-term efficacy and safety. Leveraging insights from this study, we plan to embark on a multicenter, large-scale clinical investigation to augment the robustness of the findings. Subsequent subgroup analyses will be executed, and conditions permitting, the integration of aspirin resistance gene testing, inspired by the CHANCE2 study, will be contemplated to conduct a more refined clinical investigation.

In summary, our study suggests that indobufen combined with clopidogrel is more effective and safer than aspirin combined with clopidogrel in patients with minor stroke or high-risk TIA.

## Data Availability

The datasets used and/or analysed during the current study available from the corresponding author on reasonable request.

## Declarations

### Register

www.chictr.org.cn;ChiCTR2100043087;04/02/2021.
